# YoNet: A Neural Network for Yoga Pose Classification

**DOI:** 10.1007/s42979-022-01618-8

**Published:** 2023-02-08

**Authors:** Faisal Bin Ashraf, Muhammad Usama Islam, Md Rayhan Kabir, Jasim Uddin

**Affiliations:** 1 Department of Computer Science and Engineering, University of California, Riverside, CA USA; 2 School of Computing and Informatics, University of Louisiana at Lafayette, Lafayette, LA USA; 3 Department of Computing Science, University of Alberta, Edmonton, AB Canada; 4 Department of Applied Computing and Engineering, Cardiff School of Technologies, Cardiff Metropolitan University, Cardiff, Wales, UK

**Keywords:** Pose recognition, Deep learning, Image classification, Yoga pose, Neural network

## Abstract

Yoga has become an integral part of human life to maintain a healthy body and mind in recent times. With the growing, fast-paced life and work from home, it has become difficult for people to invest time in the gymnasium for exercises. Instead, they like to do assisted exercises at home where pose recognition techniques play the most vital role. Recognition of different poses is challenging due to proper dataset and classification architecture. In this work, we have proposed a deep learning-based model to identify five different yoga poses from comparatively fewer amounts of data. We have compared our model’s performance with some state-of-the-art image classification models-ResNet, InceptionNet, InceptionResNet, Xception and found our architecture superior. Our proposed architecture extracts spatial, and depth features from the image individually and considers them for further calculation in classification. The experimental results show that it achieved 94.91% accuracy with 95.61% precision.

## Introduction

Humans are predisposed to a variety of health-related diseases due to a variety of factors such as aging, poor diet, lifestyle choices, and daily routine activities. Medical care and prescriptions for cure remain a popular mode for healthcare providers and recipients; however, the emergence of antibiotic resistance and complications to such cure lead researchers and care professionals to opt for preventive and integrative forms of medical activities and therapies for diseases rather than medicine as a supplement [1]. Daily exercises are important for human well-being, especially for older adults [2]. Extensive research has shown that physical activities with gamification and exergames as its end product play an important role in sustainable leisure activities and human well-being [3].

Yoga, as a form of integrative therapy, has gained significant traction over the years due to its unique blend of lifestyle amalgamated with exercises coupled with lifestyle choices that leads to a unique way of aging and well-being for people of all ages [4]. Researchers have found that, yoga plays an important role in the proper function by disciplining the physical and mental attributes, giving significant control over the body and mind. Stress, anxiety, flexibility, and muscle strength are just a few examples out of the many areas where yoga has shown significant benefits [4].

Pose detection is explored in  [5] where accelerometer sensors were amalgamated with micro-controllers for detecting the poses. Garg’s team [6] utilized CNN and codified the conceptual skeletonization for body key point identification through MediaPipe library for yoga pose classification that is helpful for real-time classification. Real time recognition of yoga poses with a similar concept of computer vision was employed in [7]. Human posture detection in general with a hybridized approach through amalgamating inceptionV3 and SVM was seen to be performed in Ogundokun’s work [8].

Yoga is typically practiced at home or in a training center setting, where an expert demonstrates the steps for the participants to follow. However, the global coronavirus pandemic has altered the nature of activities in close proximity due to its lethal airborne nature. The new normal has paved the way for remote exercises using the power of technology to bring the world closer together in this unprecedented situation  [9]. Yoga as a form of exercise and lifestyle also gained popularity during this pandemic and was said to be convenient, easy, and affordable [10], which paved the way for us to devise effective yoga automation that led to further pose detection through machine learning.

In this work, we developed a deep learning-based model to identify five different yoga poses and compared it to the current standard image classification models such as ResNet, InceptionNet, InceptionResNet, and Xception architecture. With an accuracy of 94.91, our model outperformed the nearest best available architecture of 91.52 in reported performance metrics.

The paper thus is segmented below as follows. The following section, “Related Works” discusses the related works that have been done in regards to the evolution of pose detection, yoga pose detection, and where the current research is headed towards. “Model Architecture” discusses the proposed model architecture with associated functions and hyperparameter discussion and rationale behind the usage of those functions. “Experimental Analysis” puts a discussion forward based on the experimental analysis carried out in this research work, and lastly, “Conclusion” summarizes the whole work with suggestions for future works of this domain.

## Related Works

The research on human pose estimation has grown significantly with the advent of image processing and recognition in 2D and 3D space [11–13]. Chen  [11] have put forward an extensive survey outlining image-based monocular human pose estimation with the aid of Deep Learning (DL)-based techniques. They noted that various pose estimation techniques, such as human body model-based and body-free, pixel-level analysis, body joint point mapping, and heat-map mapping are currently available. The rationale for deep learning for human pose estimation is substantiated by CNN’s ability to achieve state-of-the-art results when AlexNet proved its worth by starting a revolution in image classification task that still persists today [14].

Faisal [15] extended on Chen’s research [11] regarding body-joint estimation and noted the current state-of-the-art where they iterated that the body-joint points are detected by various researchers using gyroscope and joint angle algorithms for finding joint point angle, and multiple sensor fusion.

The authors in [16] engaged in pose-based human activity estimation, repeating Faisal and Chen’s research [11, 15]. To add that template-based, generative model-based, and discriminative model-based methods are popular methods of pose estimation, which are later authenticated in human pose estimation work done in [17]. Because of its perceived contribution to positive health and wellness, yoga has grown in popularity among human pose estimation. Nagalakshmi explored the impact of yoga and found that apart from treating musculoskeletal disorders and promoting a healthy lifestyle, remote yoga exercises have gained substantial popularity after the COVID outbreak [18]. A substantial gap is identified in remote yoga pose estimation and paved the way for researchers to contribute yoga pose detection methods as separate and highly influential research in human pose estimation [18].

Agarwal [19] experimented with different Machine Learning (ML) techniques for this task. To address the issue, they prepared a dataset of 5,500 images for ten different yoga poses and then applied the tf-pose estimation algorithm to extract the skeleton images in real-time. They found random forest classifiers working better for their dataset. Liaqat et al.  [20] combined traditional machine learning approaches with deep neural networks to develop a hybrid posture recognition approach. The final class prediction is made by combining the deep learning model’s weight learned with the traditional model’s prediction. In another work [21], the authors have used OpenPose for keypoint extraction followed by a hybrid CNN-LSTM layer for classification. 88 videos for six yoga poses were used to build the model.

The use of ensemble deep models in challenging home environments of varying backgrounds is seen in research work carried out by Byeon and his team [22]. They made some interesting findings related to the applicability of exercise systems in home settings for older adults and exercising with the ease of home comfort that resonates with the ideology generated in the post-COVID era. Kulikajevas and his team [23] proposed a Deep Recurrent Hierarchical Network based on MobileNetV2 for the sitting pose estimation and achieved 91.47% accuracy. Human body movements based on sonification methods were explored in [24]. Similarly, the usage of SVM and boosting for pose detection is explored in [25] and a specific concentration towards yoga was explored in Nagalakshmi’s work [26].

Redundancy of over-parameterization has been addressed through tensor-based parameterization in [27] where the researchers used the tensor-parameterized technique to produce highly compressed yet commendable accuracy for human pose estimation task. Trejo et al. [28] used Kinect for the human pose estimation task of yoga posture recognition that resonated with the task of human pose estimation carried out in [29, 30]. The model can simultaneously recognize the posture of six people in their suggested interactive system for yoga posture recognition. The AdaBoost technique was utilized to train their model. In real time, their model successfully detected several yoga positions. The complexity of our work remains formidable in nature both in terms of the complexity of the model as well as the task itself. As the subsequent related work has explored the research gap, and other factors on performance metrics related to the task, it is important to note that the idea of amalgamating convolution, specifically depthwise separable convolution along with batch normalization and pooling makes the architecture substantially complex. Arguments might be set as to whether a deep learning method is at all required for this task or not but the experimental results provided a solid footnote on the choice that the authors made. The subsequent section shall discuss the model architecture followed by the experimental analysis to provide the enormity and complexity of the task.

## Model Architecture

**Fig. 1:**
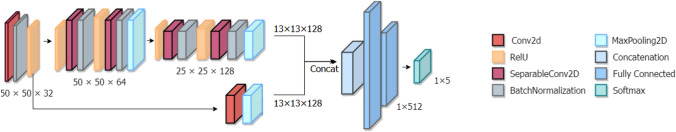
Architecture of YoNet

The proposed CNN model was inspired by the Xception model proposed by Francois Chollet [31]. The idea of depth-wise separable convolution from Xception has been incorporated into our architecture. First, a convolution layer extracts pixel features, and sequential depthwise convolution layers handle these features pixel by pixel. Depthwise convolution reduces the number of parameters and makes the computation faster. Along with the depthwise features, we concatenated the spatial features from the first layer. We added dense layers to classify the yoga poses using both depthwise and spatial features. The architecture of our proposed model is shown in Fig. 1.

We have used different types of layers in our proposed architecture. which are briefly explained here.

**Fig. 2:**
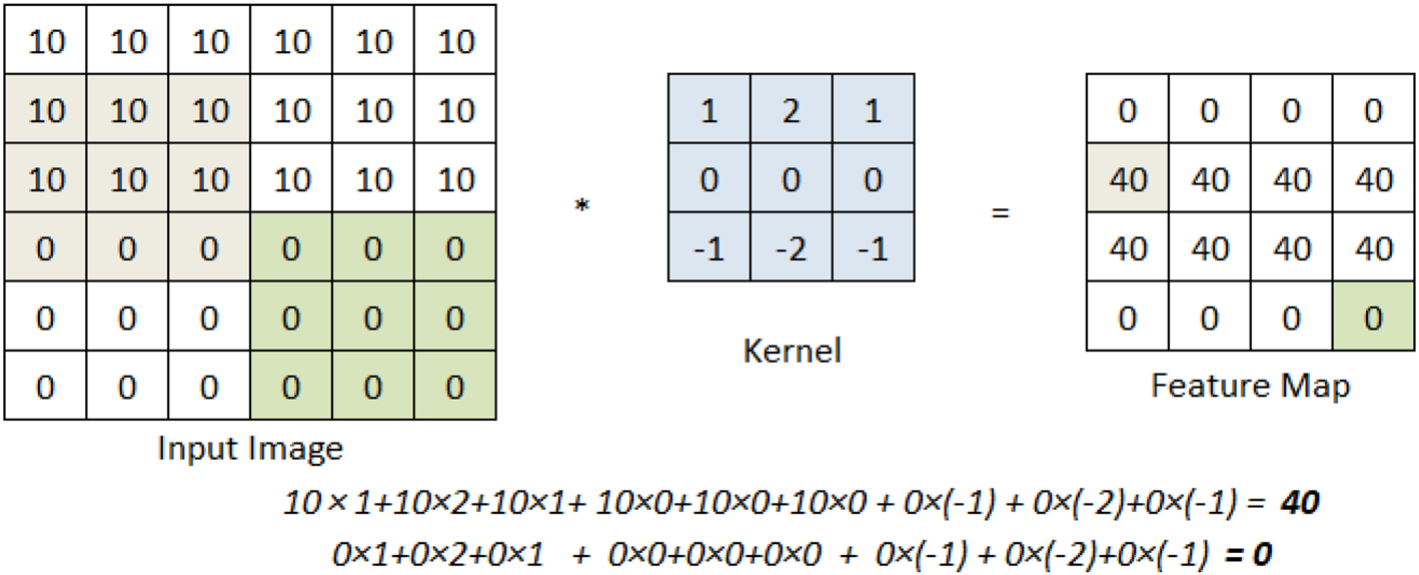
Convolution process

**Fig. 3:**
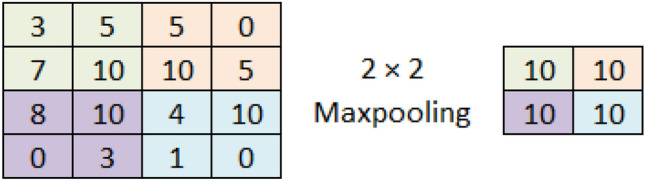
Operation of maxpooling

- Convolution: In this process, we take a small matrix of numbers, i.e., kernel, and slide it over the input image to do some matrix multiplication and transform it. We get the feature map after applying a specific kernel on the input image using the following formula (Eq. 1) where *F* and *H* denotes the input image and applied kernel respectively, *m*, *n* represents the indices of row and column of the output feature map. Figure 2 shows how a kernel is applied on a different portion of an input image, and a feature map is generated using the formula and matrix multiplications.
  1 $$\begin{aligned} O[m,n] = (F*H)[m,n] = \sum _{j} \sum _{k} H[j,k]F[m-j, n-k] \end{aligned}$$
  Our input images have three channels(RGB); hence, we applied a kernel with the same number of channels separately. The primary motivation of applying convolution is to extract meaningful information from the image. Different kernels can extract the different types of information from images such as horizontal edges, vertical edges, ridges, etc. Therefore, we have applied a different number of kernels (32, 64, 128) in different steps to extract diversified feature information.
- Batch normalization: In the training phase, the distribution of the activations in the intermediate layers may change, which causes a delay in learning as the layers need to cope with the new distribution. This problem is termed an internal covariate shift. So, we can force each layer’s input to be in the same distribution approximately by using Batch normalization. Batch normalization includes the following steps -
  1. Find mean and variance of the layer’s input using Eq. 2 and 3 respectively.
    2 $$\begin{aligned} \mu _B = \frac{1}{m} \sum _{i=1}^{m}x_i \end{aligned}$$
    3 $$\begin{aligned} \sigma _{B}^{2} = \frac{1}{m} \sum _{i=1}^{m}(x_i-\mu _B)^2 \end{aligned}$$
  2. Normalize the inputs to the layers using Eq. 4.
    4 $$\begin{aligned} \bar{x_i} = \frac{x_i - \mu _B}{\sqrt{\sigma _{B}^{2} + \epsilon }} \end{aligned}$$
  3. Scaling and shifting following Eq. 5. Here, $\gamma$ and $\beta$ are learned while training along with the parameters of the model.
    5 $$\begin{aligned} y_i = \gamma \bar{x_i} + \beta \end{aligned}$$
- Activation function: An activation function is used for getting the output which acts as the transfer function to filter the output from the layer. In this architecture, we have used the most commonly used ReLU activation function (Eq. 6).
  6 $$\begin{aligned} f(x) = max(0, x) \end{aligned}$$
- Max pooling: Max pooling reduces the dimension of feature maps from the previous convolution layer. After convolution, the feature maps can be reduced by considering only the prominent pixels in the image. Therefore, max-pooling divides the whole feature map into a given kernel size and then selects the maximum valued pixel from that window, converging dimensionality reduction. Figure 3 shows how a $2\times 2$ max-pooling reduces the dimension.

### Normal Convolution Vs Depthwise Separable Convolution

**Fig. 4:**
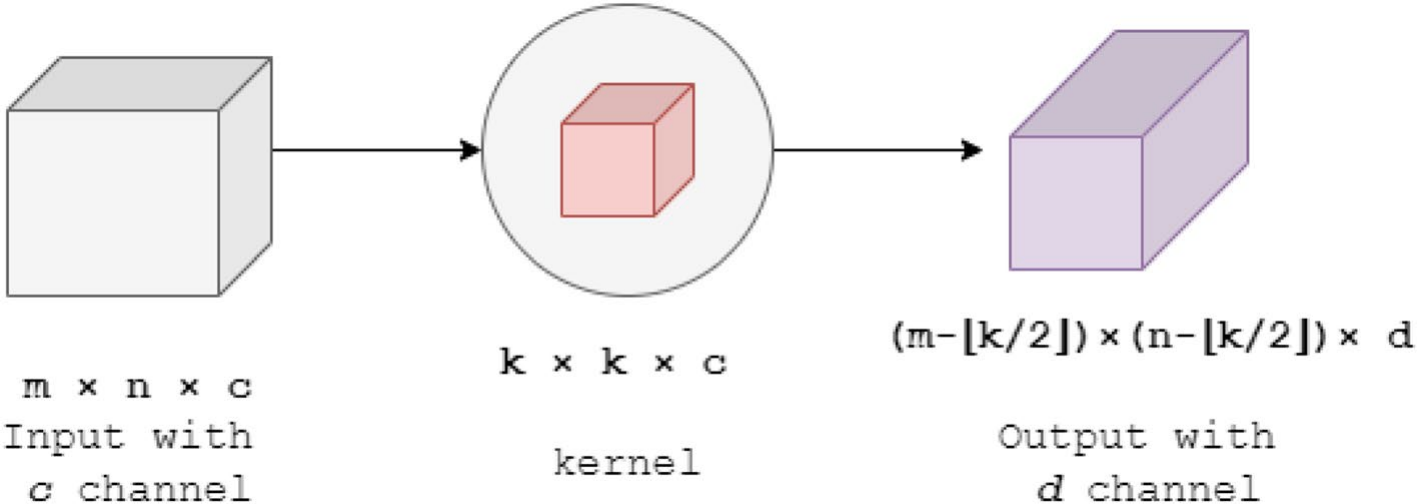
Normal convolution. $k \times k \times c$ sized kernel is applied to the input image that calculates all input channels altogether and gives the output image with one channel. If we want *d* number of channels in output, we apply *d* number of $k \times k \times c$ sized kernel and stack the output

In normal convolution, we use a kernel of size ($k \times k \times c$) to transform an image of ($m \times n \times c$). And, if we want *d* number of channels in output image, we apply *d* number of kernels and stack the outputs. Therefore, the number of multiplications needed is $(m-\lfloor \frac{k}{2}\rfloor ) \times (n-\lfloor \frac{k}{2}\rfloor ) \times k \times k \times c \times d$. Figure 4 shows the normal convolution procedure.

**Fig. 5:**
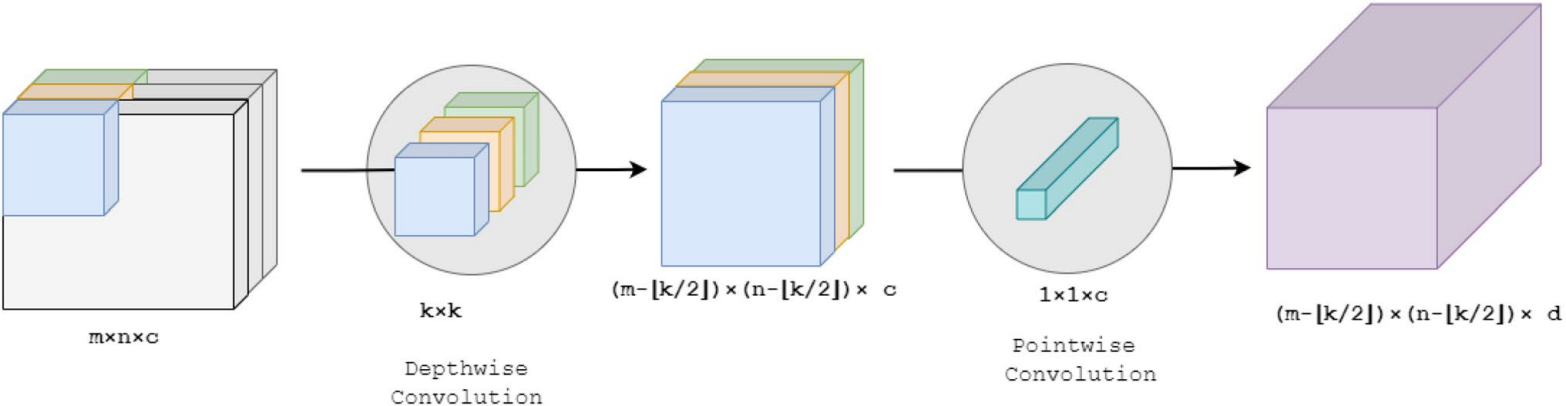
Depthwise separable convolution. First, *c* number of $k \times k$ sized kernel are used separately on *c* channels of the input image. Then, $1 \times 1 \times c$ sized kernel is used to fuse all the channels into a single channel. If we want to get d number of channels in the output image, *d* number of $1\times 1 \times c$ sized kernels are used, and outputs are stacked

In the depthwise separable convolution, we split the convolution into two processes—depthwise convolution and pointwise convolution. In the first process, we do not change the depth of the input image, and the kernel is applied to each input channel independently. Then, we apply $1 \times 1$ kernel with a depth of the input image and get the final transformed output. Now, the number of multiplications becomes $(m-\lfloor \frac{k}{2}\rfloor ) \times (n-\lfloor \frac{k}{2}\rfloor ) \times \{c \times (k \times k \times 1) + d \times (1 \times 1 \times c) \}$. Figure 5 shows the steps in depthwise separable convolution. Therefore, we get some computational advantages. The number of multiplications gets reduced by $(m-\lfloor \frac{k}{2}\rfloor ) \times (n-\lfloor \frac{k}{2}\rfloor ) \times c \times \{(k \times k \times d) - (k \times k + d) \}$.

For example, if our input image size is $100\times 100\times 3$, kernel size is $5\times 5\times 3$ and we want 32 channels in the output, then the normal convolution will require $(100-2)\times (100-2)\times 5\times 5\times 3\times 32 = 2,30,49,600$ multiplications. Again, if we follow the depthwise separable convolution, the number of multiplications required is $(100-2)\times (100-2)\times \{3\times (5\times 5\times 1) + 32\times (1\times 1\times 3)\} = 16,42,284$ which is $92.88\%$ less than normal convolution. The reason behind the drastic reduction of computation is the reduced number of transformation. In the normal convolution, we transform the image 32 times for 32 output channels, whereas in separable convolution, we transform the image once and then elongate it to 32 channels.

With the advantage of reduced computational power, depthwise separable convolutions reduce the number of parameters and may skip some features to learn. Hence, we have used features learned from regular convolution and depthwise separable convolution through concatenation to classify the poses. It ensures that the critical features for classifications are available and learned properly in the model.

## Experimental Analysis

We divided our experimentation into two parts. First, we applied some state-of-the-arts architectures extended with dense layers to classify into five classes. Then, from the idea of Xception architecture, we applied our proposed architecture to the same data and environment. In this section, we will discuss both parts separately.

### Dataset

**Fig. 6:**
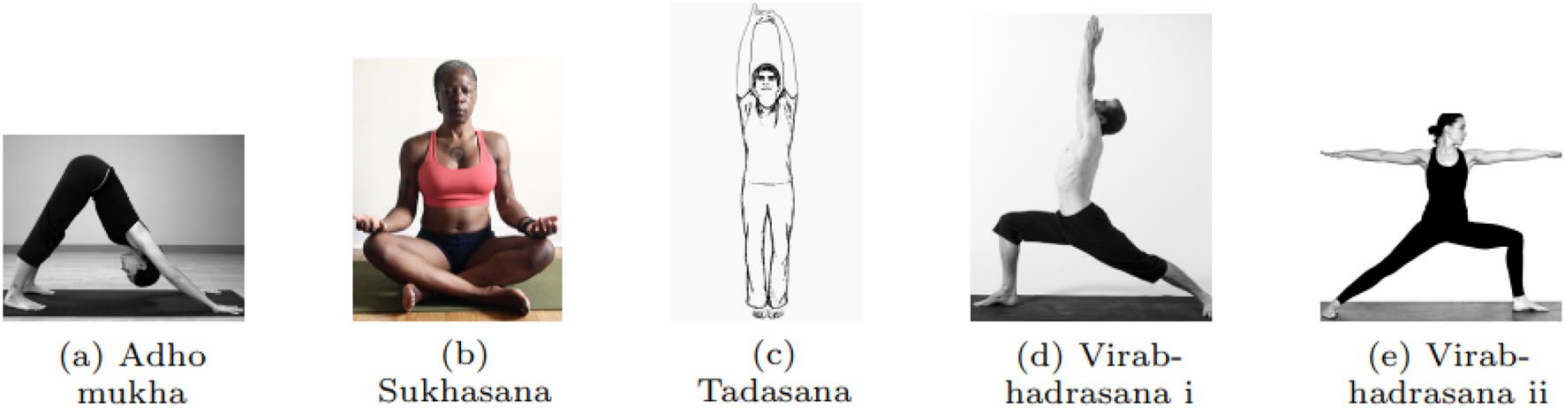
Yoga Poses various classification experiments shown in (**a**)–(**e**)

We have used the Yoga-82 dataset [32] for implementing our proposed model. We took 5 types of yoga poses for classification–Adho Mukha, Sukhasana, Tadasana, Virabhadrasana i and Virabhadrasana ii. The poses are shown in Fig. 6. The challenge of picking these 5 poses is fewer data to learn. For these poses, there are 286 images. Therefore, our target is to design a neural network that will learn from a comparatively smaller number of images and classify different poses correctly.

This dataset was collected by Verma et al. [32] from the web using the bing search engine. Then the dataset was cleaned manually. Initially, there were much different yoga poses in the dataset. Those poses are then clustered together to form a superclass. All the images in the dataset are assigned to five superclasses that we mentioned before. Our experiment in this study is to train the model to detect the superclass of the yoga pose.

Necessary preprocessing was done for each image to have the same dimension with RGB channels. Afterward, random images were split into training and validation sets. It was ensured that both training and validation sets had examples from all five classes.

### State-of-the-Art Performance vs YoNet Performance

Table 1 shows the accuracy, precision, recall, and f1-score for some state-of-the-art architectures alongside YoNet to solve the mentioned classification problem with the mentioned data. After repeating the experiment 20 times, We have noted the best performance of Resnet50, Inception V3, Xception, and Inception-Resnet V2 architectures, which are well known for image classification and have different depths and parameters. We experimented with our proposed architecture on the same dataset and in the same environment. YoNet model has a depth of 20 only with 22 M parameters. We repeated our experiments to see any anomalies and found that our architectures can classify with 94.91% accuracy.

**Table 1:** Performance of state-of-the-art architectures alongside YoNet

Architecture	Depth	#Params	Acc.	Precision	Recall	*F*1
Resnet 50 [33]	50	5,751,813	91.52	91.82	91.52	91.48
Inception V3 [34]	159	23,966,885	86.44	90.05	86.44	87.14
Xception [31]	126	23,025,581	89.83	90.21	89.83	89.79
Inception- Resnet-V2 [35]	572	55,976,549	81.35	81.64	81.35	81.29
YoNet	20	22,227,493	94.91	95.61	94.91	94.90

Best metrics from 20 repeated experiments are reported

**Fig. 7:**
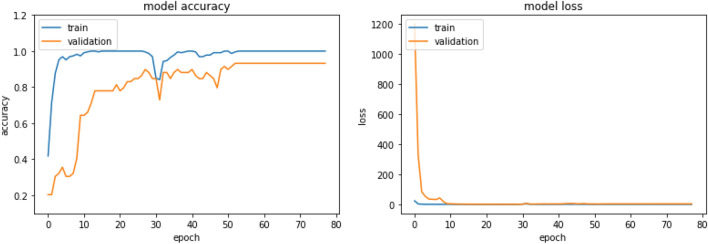
Learning curve of YoNet

From Table 1, we can see that increasing depth and parameters in the state-of-the-art architectures increases accuracy. However, increasing depth or parameters requires more computational power and time. Therefore, keeping both constraints—increasing performance and reducing computational power—we proposed our YoNet architecture. Xception architecture showed a good performance on this dataset, and so, we got the motivation from Xception architecture about depthwise separable convolution. Figure 7 shows the learning curve of our proposed model.

**Fig. 8:**
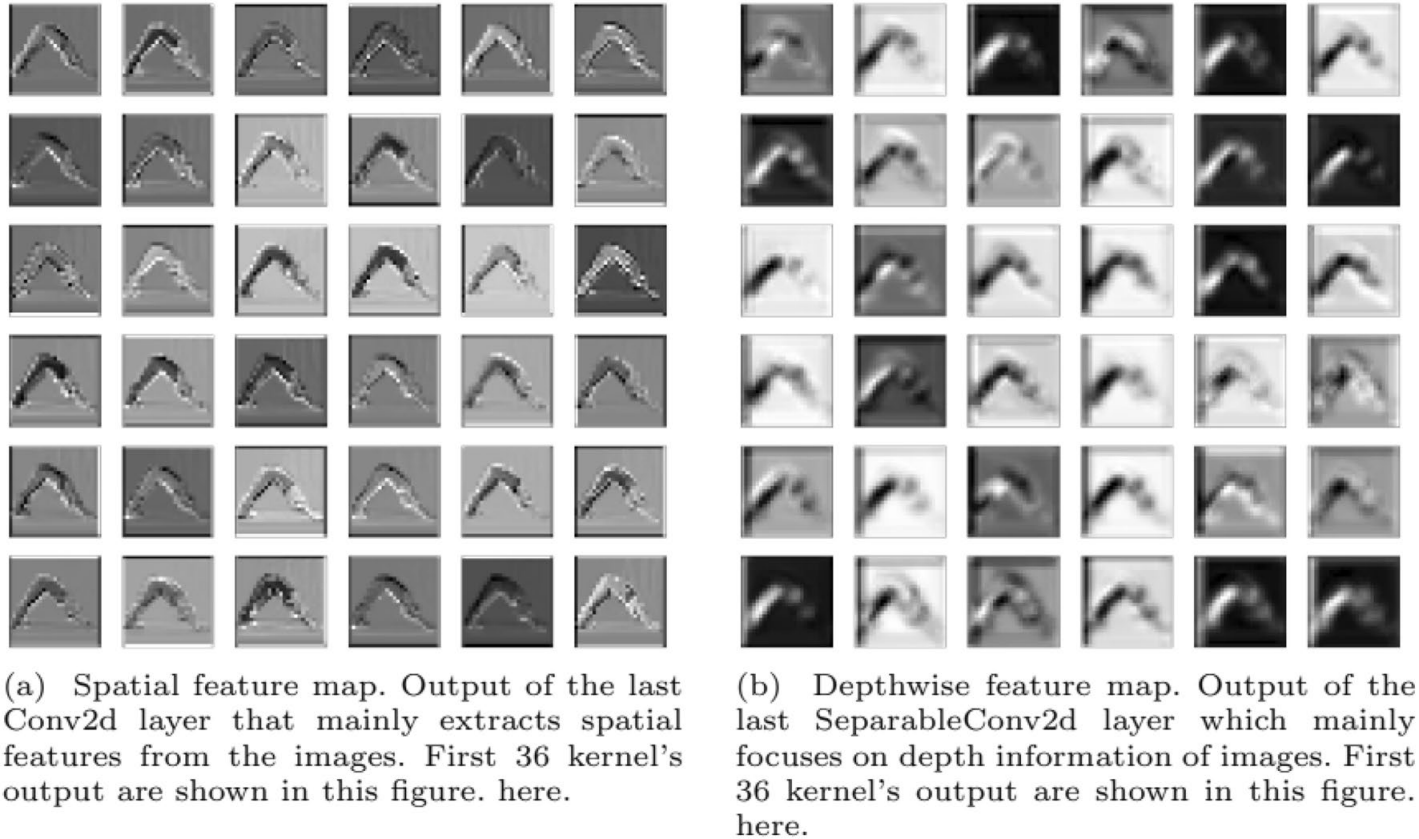
Spatial in (**a**) and depthwise in (**b**) feature map shows corresponding to “Adho Mukha” pose in yoga classification task

The fundamental motivation of YoNet architecture is to combine both spatial and depth information of the images to make the classification decision. For that purpose, we have used two types of convolution separately, which are concatenated later before classification. Therefore, we were curious to find the feature map for both merged types of convolution. Figure 8 shows the feature map for the yoga position - “Adho Mukha” from two different layers of our model. Figure 8a shows the spatial feature map from the last convolution layers where we can see different channels finding the spatial features, i.e., edges, with different kernels. Figure 8b shows the depthwise feature map from the last depthwise separable convolution layer that calculates the depth information in each pixel of the image. For other poses, we also have found a similar output. We can identify the edges and essential details from the spatial feature mapping. Depthwise feature mapping gives the position of the human body and depth information, efficiently classifying different poses, even with fewer images. Hence, our proposed architecture outperforms other state-of-the-art architectures for this given scenario.

### Statistical Analysis

We conducted a statistical analysis on the performance, in terms of accuracy, to find out whether the improvement in the accuracy that we observed is statistically significant or not. For this, we conducted 20 runs of the algorithm YoNet, Resnet 50, Inception V3, Xception, and Inception-Resnet V2. The following equation calculates the difference:

7 $$\begin{aligned} \delta =\mathcal {M}_{YoNet}({\text {test\_data}})-\mathcal {M}_{i}({\text {test\_data}}) \end{aligned}$$

Here the $\mathcal {M}$ defines the model, and the subscript i stands for different models. We measured the differences of accuracies for multiple runs for each of the architectures with the accuracy of YoNet. Then, we performed paired t-test, which is inspired by the work of Rotem et al. [36]. In algorithm 1, we showed the procedure to calculate the *p* value with the accuracy of YoNet and Resnet. We followed the same strategy to calculate the *p* value for YoNet vs. other architectures.
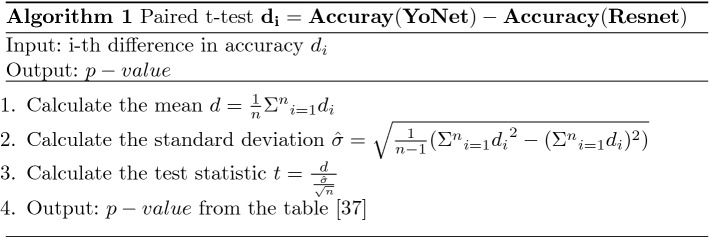

**Table 2:** *p* values to find statistical significance on the accuracy improvement of YoNet

Model pair	*p* value
YoNet vs Resnet 50	0.0110
YoNet vs Inception v3	0.0023
YoNet vs Xception	0.0001
YoNet vs Inception-Resnet V2	0.0066

We have paired t-test of our results and calculated the *p* values for each pair of architectures for detecting yoga poses. The *p* values are provided in Table 2. For each case, we see the *p* value is less than 0.05. Therefore, with at least 95% confidence, we can say that YoNet can perform better than the state-of-the-art architectures mentioned in Table 2 to classify the yoga poses.

## Conclusion

Human pose detection has been a challenging task in computer vision research for its vast and diverse application in daily life. Therefore, yoga pose recognition has immense importance for its impact on human well-being. In this work, we have proposed a novel neural network architecture, YoNet, to recognize five common yoga poses after having a thorough discussion on current related works. The intuition of our architecture is to extract the spatial and depth features from the image separately and use both types of features for recognition. It gives our architecture an advantage to differentiate better among the poses as hypothesized in our methodology and proven through result analysis and comparison carried out in our research work.

More poses can be considered even with our proposed architecture due to its strategy of extracting features. Future research work also includes better performance through hyper-parameter tuning. In conclusion, our contribution added substantial value to ongoing yoga and human pose detection with a future direction to the researchers in this area to successfully advance this paradigm to near-perfect metrics of performance that would be beneficial to all the stakeholders involved.

## Data Availability

Data sharing not applicable to this manuscript as no datasets were generated during the current study.
